# Unravelling the alkali transport properties in nanocrystalline A_3_OX (A = Li, Na, X = Cl, Br) solid state electrolytes. A theoretical prediction

**DOI:** 10.1039/d2ra03370d

**Published:** 2022-07-11

**Authors:** Long Van Duong, Minh Tho Nguyen, Yohandys A. Zulueta

**Affiliations:** Laboratory for Computational Molecular and Materials Sciences, Science and Technology Advanced Institute, Van Lang University Ho Chi Minh City Vietnam duongvanlong@vlu.edu.vn; Faculty of Applied Technology, School of Engineering and Technology, Van Lang University Ho Chi Minh City Vietnam; Institute for Computational Science and Technology (ICST) Ho Chi Minh City Vietnam; Departamento de Física, Facultad de Ciencias Naturales y Exactas, Universidad de Oriente Santiago de Cuba CP- 90500 Cuba yzulueta@uo.edu.cu

## Abstract

Transport properties of the halogeno-alkali oxides A_3_OX (A = Li, Na, X = Cl, Br) nanocrystalline samples with the presence of ∑3(111) grain boundaries were computed using large-scale molecular dynamic simulations. Results on the diffusion/conduction process show that these nanocrystalline samples are characterized with higher activation energies as compared to previous theoretical studies, but closer to experiment. Such a performance can be attributed to the larger atomic density at the ∑3(111) grain boundary regions within the nanocrystals. Despite a minor deterioration of transport properties of the mixed cation Li_2_NaOX and Na_2_LiOX samples, these halogeno-alkali oxides can also be considered as good inorganic solid electrolytes in both Li- and Na-ion batteries.

## Abstract 2

Transport properties of the halogeno-alkali oxides A_3_OX (A = Li, Na, X = Cl, Br) nanocrystalline samples with the presence of ∑3(111) grain boundaries were computed using large-scale molecular dynamic simulations.

## Graphical abstract

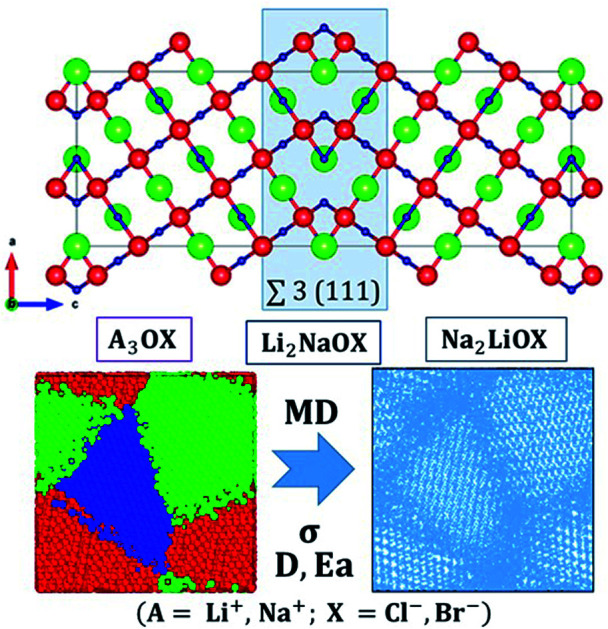

## 1. Introduction

The search for affordable inorganic electrolytes for future all solid state alkali-ion batteries is one of the current hot topics due to the urgent need for high energy density materials and relevant safety issues.^1–5^ Exploration and prediction of the properties of electrolytes are crucial for the improvement of performance and durability of the battery.^1–7^ Of particular interest, the development of inorganic solid electrolyte faces critical issues, such as the lower electrical conductivity as compared to conventional liquid electrolytes, interfacial resistance with the electrodes, narrow electrochemical window that constrain its practical applications.^4–7^

Of the compounds with special interest for inorganic solid electrolyte, the Li-rich anti-perovskite Li_3_OX (X = Cl^−^, Br^−^) has attracted large scientific interest due to the high Li ionic conductivity (≈1 mS cm^−1^) and stability with the electrode, and low Li activation energy (≈0.2 eV).^8–12^ Nevertheless, a higher activation energy (≈0.6 eV) and a lower ionic conductivity for Li_3_OX have recently been reported experimentally.^12–14^ Further studies concerning the composition screening of Li and Na anti-perovskite structure were performed disclosing the defect chemistry and ionic transport in pristine, Li_3_OCl_1−*y*_Br_*y*_ and Li_3−*x*_Na_*x*_OCl_1−*y*_Br_*y*_ compositions.^15–18^ For instance, in mixed anionic samples the Cl/Br mixing have small contribution to the conducting properties as compared to the pristine samples, but this doping strategies can be used for fine tuning the transport properties in such anti-perovskite structures.^16^

Dawson *et al.*^15^ reported the ionic transport at stable grain boundaries of Li_3_OCl as a model polycrystalline electrolyte. These authors predicted high concentrations of grain boundaries and disclosed that the Li-ion conductivity is lower through the grain boundaries with higher activation energies for Li-ion conduction than that of the bulk crystal, thereby confirming the high grain boundary resistance in this material.^15,17^ The vast contribution of the grain boundary to the overall conducting properties of Na_3_OBr and Li_3_OCl was reported,^12,15^ and further confirmed by theoretical studies taking the grain boundaries into account.^15,17,19^

In a solid polycrystal, a grain boundary is a planar defect dividing two grains with different crystallographic orientation. According to the coincidence lattice site theory, two independent grains are tilted by an angle until the individual surface plane coincides, resulting in the desired grain boundary ∑(a,b,c) where ∑ denotes the coincidence index and (a,b,c) the grain boundary plane.^15,17^ Despite earlier studies concerning the transport properties of individual grain boundaries and bulk A_3_OX materials,^15,17^ the explicit influence of the grain boundaries in polycrystalline samples has not been reported yet. Besides, the ∑3 grain boundary type has the lowest segregation energy in compounds such as inverted perovskite structures.^15,19^ The ∑3 grain boundary type is thus expected to appear with high density in real nanocrystalline samples due to their low energy, but the role of this grain boundary remains almost unexplored.

Motivated by the above considerations, we set out to investigate in the present study the influence of the ∑3(111) grain boundary type on the transport properties in A_3_OX (A = Li^+^, Na^+^, X = Cl^−^, Br^−^) by using large-scale molecular dynamics computations. Alkali diffusion in nanocrystalline A_3_OX samples, which is of vital importance for understanding of the role of grain boundary, is investigated. This study also includes the case of intercalated samples such as Li_2_NaOX and Na_2_LiOX with the aim to elucidate their capabilities as inorganic solid electrolytes for future Li and Na solid state batteries. The main difference of the present results with respect to the previous studies consists in that we are disclosing the Li and Na transport properties of pristine A_3_OX and Li_2_NaOX and Na_2_LiOX nanocrystals, with the presence of the ∑3(111) grain boundaries, to simulate the insertion/disinsertion process in the polycrystalline A_3_OX solid state electrolytes.

## 2. Computational protocols

**Fig. 1:**
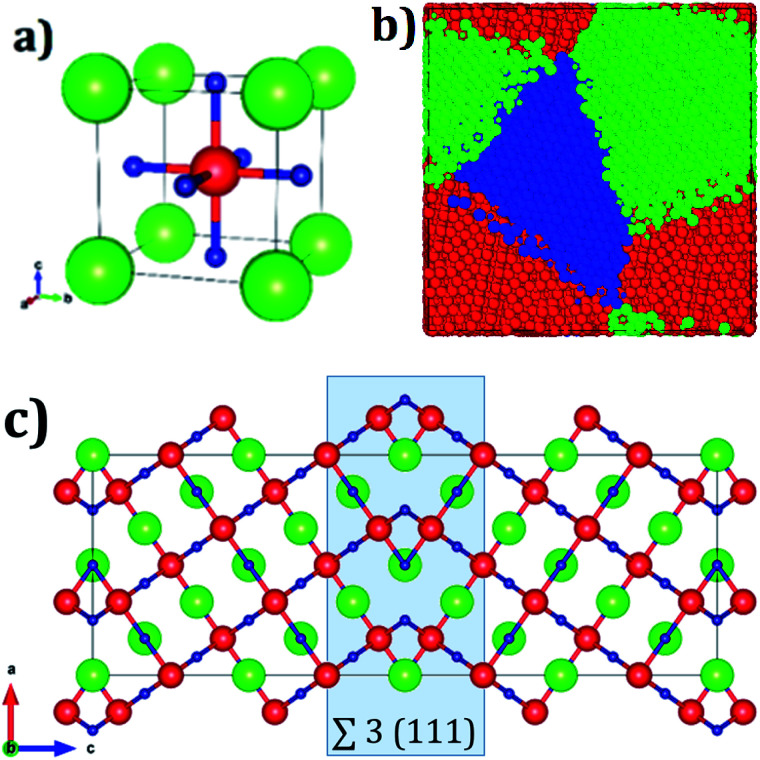
(a) Unit cell of A_3_OX (A = Li^+^, Na^+^, X = Cl^−^, Br^−^) lattice structures, green, red and blue represents X^−^, O^2−^ and A^+^ ions respectively, (b) A_3_OX nanocrystal with three grains, and (c) ∑3(111) grain boundary, where the shaded square highlights the grain boundary region. Color coding in (b) represents a unique grain.

Fig. 1a displays the conventional representation of the A_3_OX (A = Li^+^, Na^+^, X = Cl^−^, Br^−^). In this particular anti-perovskite structure with space group *Pm-3m* No. 221, the body center is occupied by oxygen, whereas the eight vertexes by the X^−^ anions and the six face centers are occupied by the A^+^ alkali ions.^15,17,18^ The lattice parameters *a* for Li_3_OBr and Na_3_OBr are 3.981 and 4.541 Å, respectively, whereas values for Li_3_OBr and Na_3_OBr amount to 3.991 and 4.613 Å, respectively.^15,17–20^ Following the coincidence site theory, the symmetric tilt ∑3(111) grain boundary,^15,19^ depicted in Fig. 1c, is construed from the A_3_OX (A = Li^+^, Na^+^, X = Cl^−^, Br^−^ in an ionic form) lattice structures.

Nanocrystalline A_3_OX samples are constructed employing the Voronoi tessellation method implemented in the Atoms code.^21^ In this method, samples with three grains with average grain volume of 17.20 nm^3^ are built by filling in a cubic box with randomly distributing seeds. We use the ∑3(111) grain boundary as the seed for generating the samples, considering the fact that the resulting nanocrystals are constituted by three grains connected with a ∑3(111) grain boundary.

The simulation cubic box has a size of 80 × 80 × 80 Å^3^, containing 42 945 atoms with ionic compositions of 25 780 A^+^, 8615 O^2−^ and 8550 X^−^. In order to eliminate free surface effects, the periodic boundary conditions are imposed in all directions. Therefore, the overlapped atoms within 1.5 Å distances are separated in 0.2 Å and double atoms are deleted to eliminate the initial unwanted artificial defects which may arise from the tessellation process. The extra net charge introduced by the tessellation process is compensated by the A^+^ and X^−^ vacancies generated randomly. Inclusion of A^+^ and X^−^ vacancies is in line with the predicted alkali halide partial Schottky defects, leading to an A^+^ vacancy migration mechanism, which is the most favorable scheme with low formation energy.^11,16^ These pair defects can be expressed according to Kröger–Vink notation by eqn (1):1
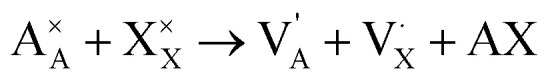
where 
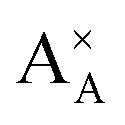
 and 
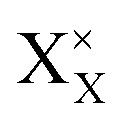
 denote the A^+^ and X^−^ ion in their lattice position, whereas 
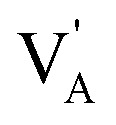
 and 
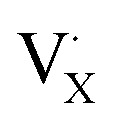
 represent a A^+^ and X^−^ vacancy, respectively, and AX the alkali halide. In eqn (1), for each A-vacancy, one X-vacancy is generated for charge compensation resulting in a stoichiometric A_3−*y*_OX_1*−y*_ formula. As mentioned above, eqn (1) is used during the construction of the simulation boxes, resulting in a representative defect concentration of *y* = 0.118. Note that the nanocrystalline A_3_OX structures inherently exhibit a conduction mechanism *via* an alkali vacancy (
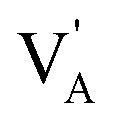
) as described in eqn (1).

The resulting polycrystalline samples A_3_OX with A = Li^+^, Na^+^, X = Cl^−^, Br^−^ are depicted in Fig. 1b. For large-scale molecular dynamics simulations (MD) the LAMMPS code is used to determine the Li^+^ and Na^+^ diffusion data of nanocrystalline A_3_OX as each can be split in terms of ions A = Li^+^, Na^+^ and X = Cl^−^, Br^−^ samples.^22^ The potential parameters reported in ref. 16 are adopted to model the particle interactions. These potential parameters consider the pure electrostatic interactions for long-range together with a Buckingham-type potential for the short–range pair ion interactions. Further details concerning the potential parameters can be found in ref. 16. In addition, formal valence charge is used for all the species in each nanocrystal. The simulations are performed in a temperature (*T*) range of 500–1200 K each step of 100 K. The simulation boxes are first relaxed using an isothermal-isobaric ensemble (NTP) for reaching the thermodynamic equilibrium; the production runs are carried out with an isothermal-isochoric ensemble (NVT) recording the mean square displacement (MSD) for the A^+^ ions. The diffusion coefficients (*D*) are obtained from the MSD plots by eqn (2):

2 MSD = 6Dt

where *t* is the simulation time. Each production run is limited to 2 ns with a time step of 2 fs.

The ionic conductivity or direct current conductivity (*σ*_DC_) can be obtained employing the Nernst–Einstein formulation (3):

3 *σ*(*T*) = *H*_V_*Nq*^2^*D*(*T*)/*k*_B_*T*

where *q* is the charge of the mobile ion, *N* the charge density of the mobile ion, *k*_B_ the Boltzmann constant, and *H*_V_ (=1) represents the Haven's ratio used to consider the effect on the mobility of the charge carrier (ion) of the external electric field in real samples.^23–25^ Following application of this procedure for various temperature values, we obtain a series of values for *D*(*T*) and *σ*(*T*), that can be fitted with the aid of an Arrhenius-type function (4):

4 *D*(*T*) = *D*_0_ exp(−*E*a/*k*_B_*T*); *σ*(*T*) = *σ*_0_ exp(−*E*a/*k*_B_*T*)

to obtain the simulated activation energy (*E*a) for diffusion and electrical conduction, respectively. In eqn (4), *D*_0_ and *σ*_0_ are pre-exponential factors for diffusion and conduction, respectively. The simulated values of *D*(*T*) and in particular of *σ*(*T*)are valuable parameters as they can be directly compared to the experimental results reported in the literature.^6,15,23–25^

## 3. Results and discussion

### 3.1 Alkali ion migration in A_3_OX (A = Li^+^, Na^+^, X = Cl^−^, Br^−^) nanocrystals

**Fig. 2:**
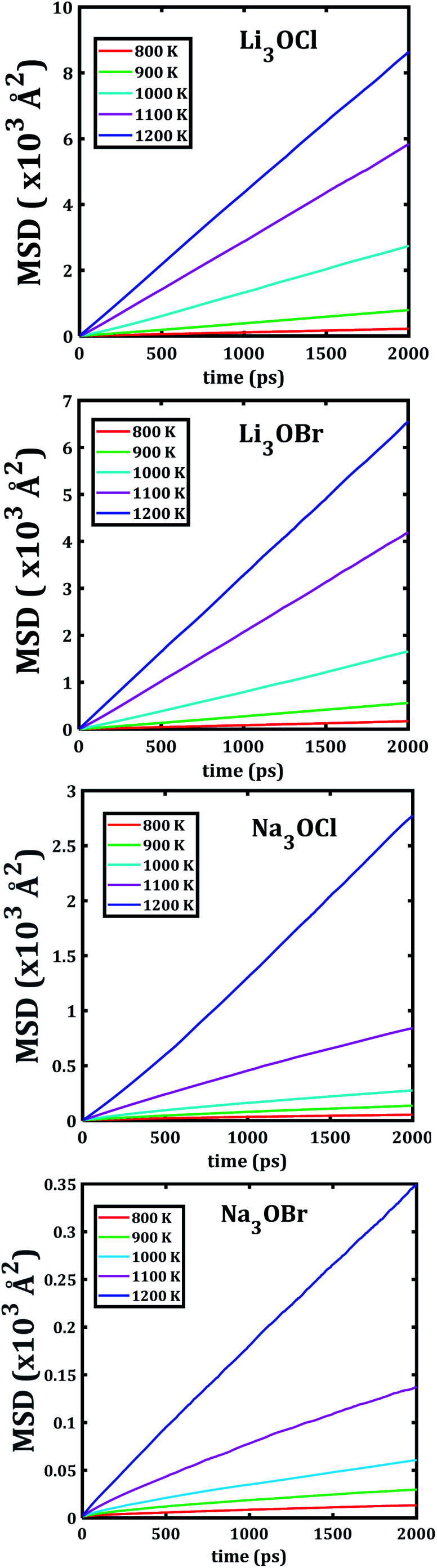
Mean square displacement *versus* simulation time of each A_3_OX nanocrystalline sample.

The temporal evolution of the MSD values of each A_3_OX nanocrystal is illustrated in Fig. 2. In all cases, the MSD dependence is clearly linear, and the slope increases upon increasing temperature, indicating a long-range alkali atom migration within the respective nanocrystalline structure. It is noteworthy that the Li_3_OX samples have higher values of MSDs as compared with Na_3_OX counterparts. Therefore, the order of MSDs in Li_3_OCl is similar to that for Li_3_OBr. On the contrary, a difference of the first-order of MSDs is observed in Na_3_OCl and Na_3_OBr samples. This is attributable to the difference between the ionic radius and atomic mass of the Li^+^/Na^+^ ions. In addition, the lattice parameters of Na_3_OX lattice structure are larger than those of Li_3_OX,^16,20^ and consequently the jump distance is larger in Na_3_OX structures as compared with Li_3_OX limiting the ease of Na-ion migration in Na_3_OX structures *via* a Na^+^ vacancy mechanism.

**Fig. 3:**
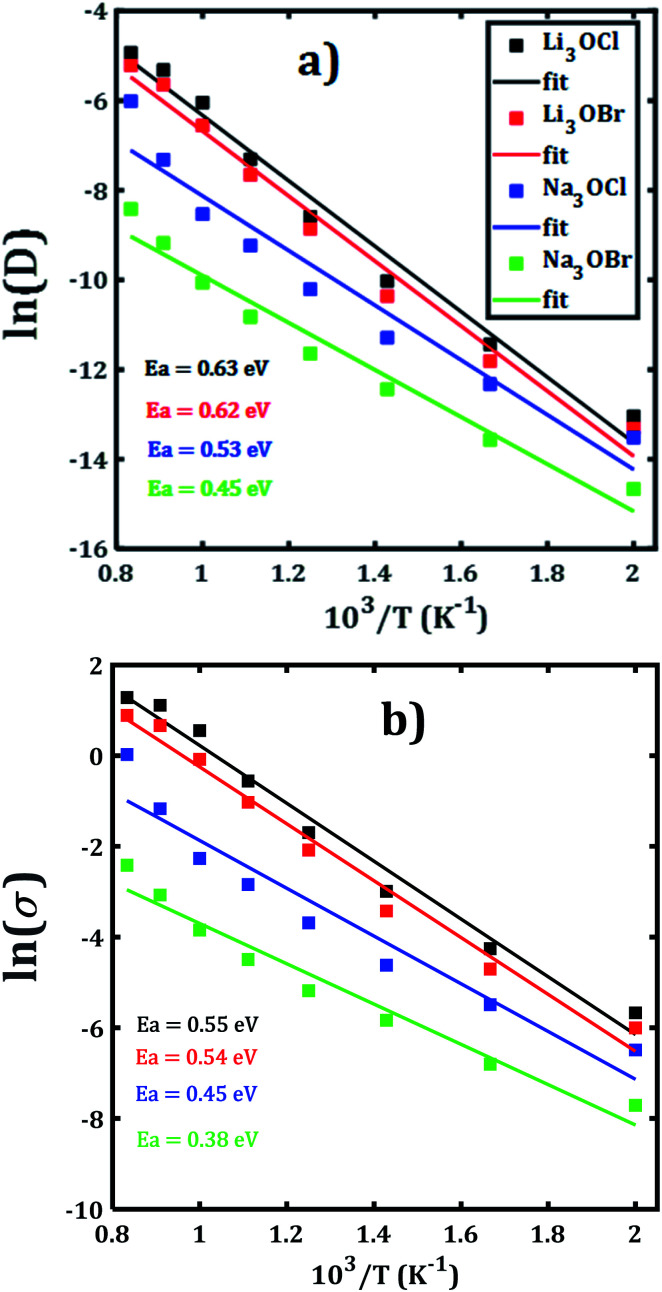
Arrhenius dependence of (a) diffusion coefficient and (b) DC conductivity of each A_3_OX nanocrystalline samples.

Alkali diffusion coefficient and conductivity at each temperature for each A_3_OX nanocrystal are obtained from eqn (2) and (3), respectively. The Arrhenius-type dependence of both diffusion coefficient and conductivity is depicted in Fig. 3. Table 1 compiles the results of activation energy for diffusion and conduction, including the interpolated values at 300 and 500 K of conductivity of A_3_OX nanocrystals (*σ*(300 K) and *σ*(500 K), respectively). The Li_3_OCl samples have the largest value of conductivity, followed by Li_3_OBr, Na_3_OCl and Na_3_OBr in a decreasing order. This behavior was previously reported by Dawson *et al.* in monocrystalline samples (*i.e.* without grain boundaries).^16^ The activation energy for diffusion is consistently lower as compared to that for the conduction, indicating the existence of non-efficient jumps contributing to the conductivity, despite the difference between mass and charge transport phenomenon.

**Table 1:** Transport properties of A_3_OX antiperovskite structures. Ea^D^ and Ea^σ^ (in eV) represent the activation energy for diffusion and conduction, respectively

Structure	Specie	Ea^D^	Ea^σ^	*σ*(300 K)	*σ*(500 K)
Li_3_OCl	Li	0.63	0.55	4.35 × 10^−7^	2.13 × 10^−3^
Na_3_OCl	Na	0.53	0.45	7.12 × 10^−7^	7.90 × 10^−4^
Li_3_OBr	Li	0.62	0.54	3.44 × 10^−7^	1.47 × 10^−3^
Na_3_OBr	Na	0.45	0.38	7.77 × 10^−7^	2.9 × 10^−4^

Hereafter, the results are discussed on the basis of activation energy for conduction; a similar discussion can be followed for the diffusion process. The lowest values of activation energy are obtained for Na_3_OCl and Na_3_OBr, being 0.45 and 0.38 eV, respectively, whereas the activation energy amounts to 0.54 eV for Li_3_OCl and Li_3_OBr. These values are larger as compared to those reported in ref. 16 for monocrystalline samples. The conductivity at 500 K reaches the value of 1.47 and 2.13 × 10^−3^ Scm^−1^, for Li_3_OBr and Li_3_OCl, while 2.9 and 7.9 × 10^−4^ Scm^−1^ for Na_3_OBr and Na_3_OCl, respectively.

With respect to the activation energies and conductivity at 500 K reported in early studies, clearly our present calculated values are quite different, with larger activation energies and slightly lower conductivities. Considering the reported underestimation of calculated values for transport properties (*i.e.* activation energy, diffusion coefficient and conductivity) for these compounds,^15–17^ the inclusion of the polycrystalline nature with presence of ∑3(111) grain boundary makes the results more credible. In general, the present trend and magnitude of the conductivity are more consistent with available experimental data. For instance, the bulk Li_3_OCl is characterized with an activation energy of 0.54–0.59 eV and a conductivity of 5.8 × 10^−7^ Scm^−1^.^12,17,25,26^ A recent theoretical study of the Na^+^ migration in Na_3_OBr which was based on a combined deep potential MD, *ab initio* MD and static simulations, revealed a diffusion activation energy of 0.41–0.43 eV and a conductivity at ambient temperature up to 2 × 10^−7^ Scm^−1^,^27^ even a static simulation study reported an activation energy as low as 0.34 eV.^17^ Earlier study of transport properties of Na_3_OCl obtained a conductivity at 500 K of 2 × 10^−4^ Scm^−1^.^16,27^ From the results listed in Table 1 we can conclude on a consistency between the reported findings^15,16^ and our present results. In particular, the transport properties reported in the present paper are more realistic considering the presence of ∑3(111) grain boundaries and polycrystalline nature in such A_3_OX structures. Our results explicitly confirm the fact that the presence of grain boundary in the computations, particularly the ∑3(111) entails the high grain boundary resistance in these compounds.^15,17^

**Fig. 4:**
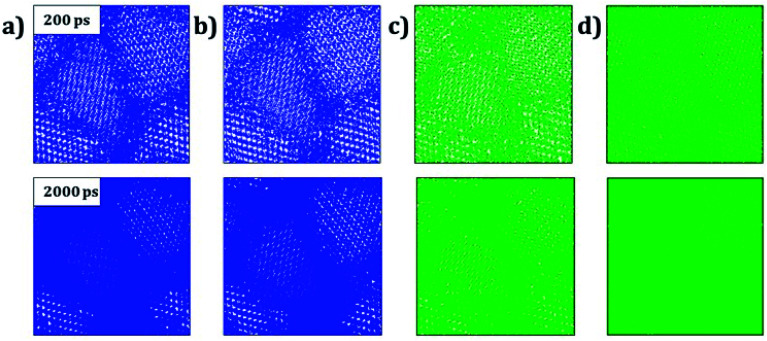
Trajectory density map of (a) Li_3_OCl, (b) Li_3_OBr, (c) Na_3_OCl and (d) Na_3_OBr compounds after 200 ps (top images) and 2000 ps (bottom images). Blue and green lines represents the Li and Na trajectory lines, respectively.

Trajectory density plot is a powerful tool to visualize the ion mobility, especially in polycrystalline materials.^15,24^Fig. 4 depicts the Li and Na trajectory plot at 200 ps of A_3_OX nanocrystals. In all cases the trajectory maps confirm the 3D characteristic of alkali migration. At 200 ps, the granulated samples are clearly visible except for Na_3_OBr sample having a higher density trajectory map, which implies that the Li and Na mobility is constrained when they are close to the grain boundaries.

The Li and Na mobility through the grain boundaries is not restricted because these conducting regions contain more migration paths, where the alkali-ion diffuses without the need of alkali vacancy as a conduction mechanism which is required for bulk A_3_OX and monocrystalline samples. With the temporal evolution, alkali ions can migrate to the grain *via* interstitial or Li/Na vacancy conduction mechanism. At 2 ns, the trajectory plots are getting denser, in particular for Li_3_OCl, Li_3_OBr and Na_3_OCl the granulated structure is still visible showing a higher density near of the grain boundary regions. The Na_3_OBr shows the highest density map covering the entire simulation box, implying that this compound has better Na transport properties as compared to the other cases considered. In addition, the trajectory plot becomes similar to that of a monocrystalline Na_3_OBr sample.

### 3.2 Li and Na transport properties in mixed cation systems

An earlier study reported the transport properties of monocrystalline samples of mixed cation Li_3−*y*_Na_*y*_OX and Na_3−*y*_Li_*y*_OX, predicting no significant improvements on transport properties as compared to those of the A_3_OX structures.^16^ Exploration of transport properties of these systems is of interest to further elucidate the capability of pristine Li_3_OX/Na_3_OX as inorganic solid electrolyte for lithium/sodium ion batteries. In this context, this section is aimed to a study on transport properties of the mixed nanocrystals of Li_3−*x*_Na_*x*_OX and Na_3−*x*_Li_*x*_OX. Nanocrystals are constructed by random substitution of Li/Na in Li_3_OX and Na_3_OX structures discussed in Section 3.1, leading a composition of 17 187 Li^+^, 8593 Na^+^, 8615 O^2−^ and 8550 X^−^, resulting in a stoichiometric formula Li_2_NaOX maintaining the defect concentration of A^+^ and X^−^ (*y* = 0.118). For the sodium counterpart Na_2_LiOX, the alkali composition is 17 187 Na^+^ and 8593 Li^+^, maintaining the same number of O^2−^ and X^−^ species. Both Li_2_NaOX and Na_2_LiOX preserve the ∑3(111) grain boundary as well as the amount and size of grain and the simulation box. Eqn (1) is also considered for the construction of the simulation boxes, leading 
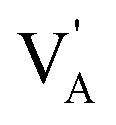
. The same setup described in Section 3.1 for large scale MD is adopted, recording the MSD for Li^+^ and Na^+^ in Li_2_NaOX and Na_2_LiOX structures.

**Fig. 5:**
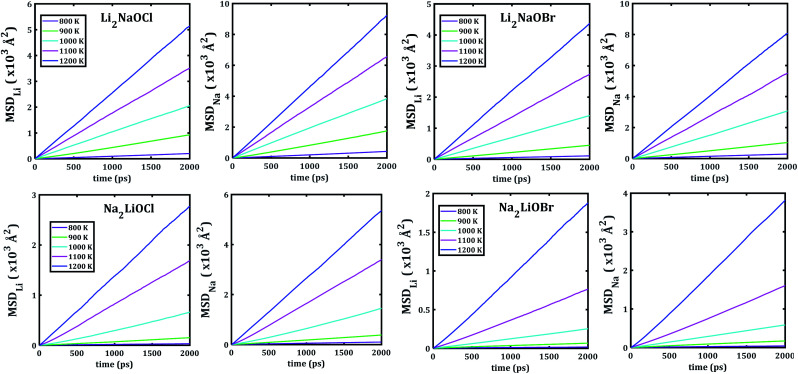
Li− and Na−mean square displacement (MSD) *versus* simulation time of Li_2_NaOX and Na_2_LiOX structures.

Fig. 5 depicts the temporal evolution of the mean square displacement of Li^+^ and Na^+^ ions in Li_2_NaOX and Na_2_LiOX nanocrystals. At shown in Fig. 5, all MSDs tend to increase the linearly with the evolution of the simulation time, and the slope increases upon the temperature change. This is an indicator for the existence of long-range diffusion.

Note that in all mixed cation samples, the order of magnitude of Na-MSD is greater than that of the Li-MSD in the entire temperature range studied, which implies better migration properties for Na^+^ ion in all samples considered.

**Fig. 6:**
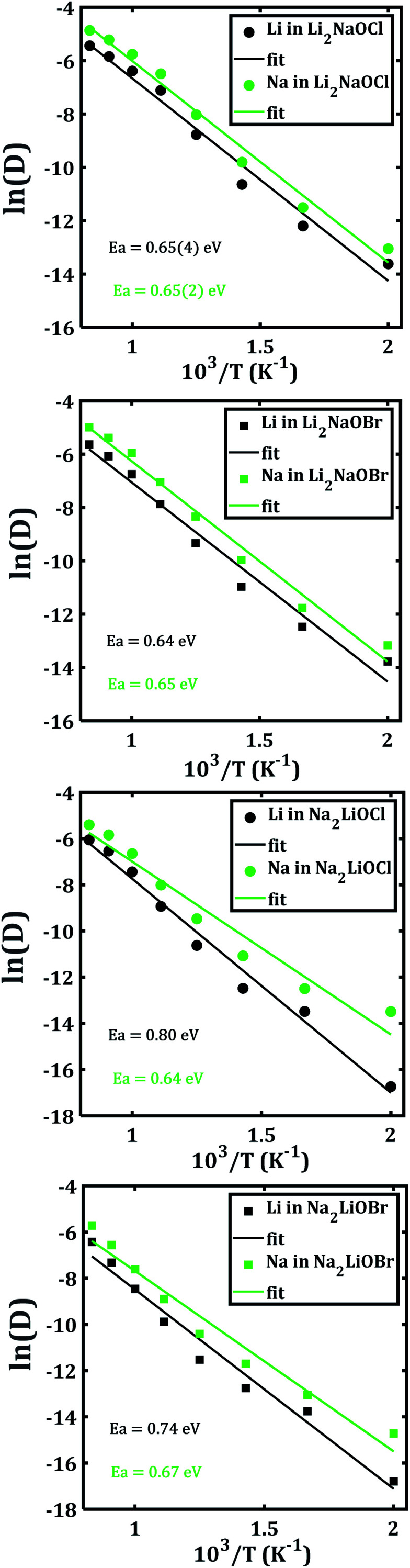
Arrhenius dependence of Li− and Na−diffusion coefficient (D) of Li_2_NaOX and Na_2_LiOX structures.

In order to disclose the migration properties, the diffusion coefficients are obtained by fitting the MSDs data using eqn (2). Fig. 6 shows the Arrhenius-type dependence of diffusion with the temperature of each mixed cation system. The straight lines in Fig. 6a and b are almost parallel, which implies similar activation energies (being ≈ 0.65 eV) for both Li^+^ and Na^+^ migrations in Li_2_NaOX systems. These activation energies for diffusion in Li_2_NaOX are similar as compared to their pristine Li_3_OX counterpart (≈0.63 eV). Surprisingly, the diffusion coefficient of Na^+^ is higher by one order of magnitude with respect to the Li^+^ diffusivity in all cases, suggesting that these systems have some potential in application as inorganic solid-state electrolyte for future Na-ion batteries. Analogously, the diffusion data are converted to the dc conductivity with the aid of eqn (4).

**Fig. 7:**
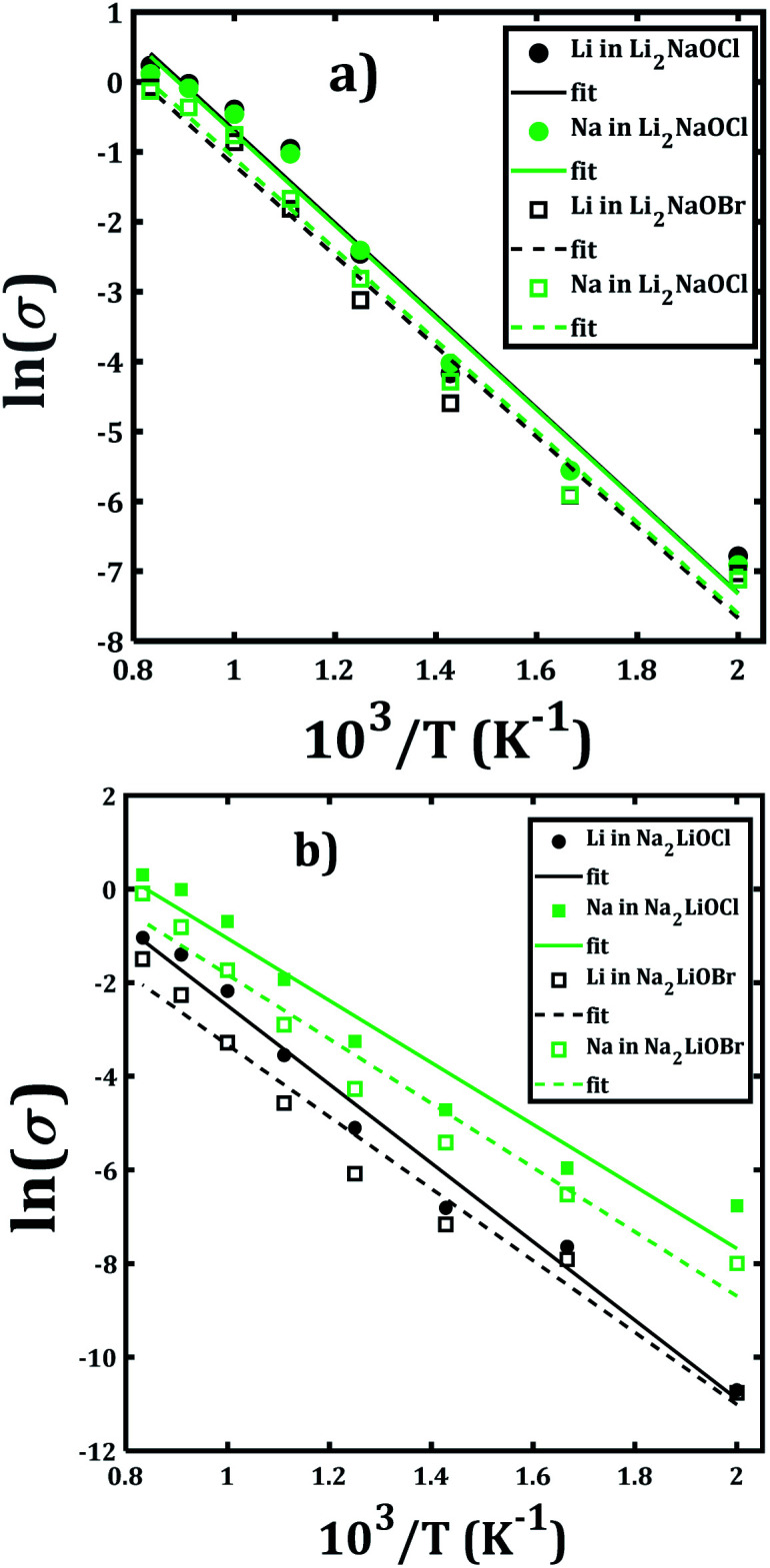
Arrhenius-type dependence of Li and Na dc-conductivity of (a) Li_2_NaOX and (b) Na_2_LiOX nanocrystalline sample.

Fig. 7 depicts the Arrhenius-type dependence of the Li^+^ and Na^+^ dc-conductivity for Li_2_NaOX and Na_2_LiOX structures. While Fig. 7a shows the case of conductivity dependence in Li_2_NaOX, Fig. 7b is related to Na_2_LiOX structures. The alkali conductivities for Li_2_NaOCl and Li_2_NaOBr structures are similar to each other (Fig. 7a). At lower temperature (<900 K) the conductivity values are higher by two, even three orders of magnitude in Li_2_NaOX with respect to the Na_2_LiOX compound, except for the Na conduction in Na_2_LiOCl structure with similar values upon temperature change.

Table 2 summarizes the activation energies for Li and Na diffusion and conduction, including the conductivity at 300 and 500 K in Li_2_NaOX and Na_2_LiOX structures. As in the case of the pristine A_3_OX compounds, the activation energy for both alkali ions diffusion is larger than that for conduction, due to non-effective jumps contributing to the long-range conductivity. Li_2_NaOX have smaller activation energies, being between 0.65/0.56 eV for diffusion/conduction, respectively. Our calculated results of conductivity at 300 K reach to the values of 1.02 × 10^−7^ and 6.62 × 10^−4^ Scm^−1^ at 500 K for Na^+^ conduction in Li_2_NaOCl nanocrystalline sample; both quantities lie well in a range for those obtained for the pristine Na_3_OCl.^16^ Our results are also consistent with the reported Li conductivity of ≈ 10^−4^ Scm^−1^ which was reported for Li_2_NaOX at 500 K.^16^ In the case of Li_2_NaOBr, the Li^+^ and Na^+^ conductivities at 300 K and 500 K are in the order of 8 × 10^−8^ and 5 × 10^−4^ Scm^−1^, respectively. For Na_2_LiOX structures, again the Na^+^ conduction suggests its better transport properties in view of their lower activation energy and higher diffusion coefficient and conductivity at 300 and 500 K. Among the Na_2_LiOX structures, Na_2_LiOCl presents us with some better Na transport properties.

**Table 2:** Transport properties of Li_2_NaOX and Na_2_LiOX antiperovskite structures. Ea^D^ and Ea^σ^ (in eV) represent the activation energy for diffusion and conduction, respectively

Structure	Specie	Ea^D^	Ea^σ^	*σ*(300 K)	*σ* (500 K)
Li_2_NaOCl	Li	0.65(4)	0.57(0)	9.86 × 10^−8^	6.68 × 10^−4^
	Na	0.65(2)	0.56(7)	1.02 × 10^−7^	6.62 × 10^−4^
Li_2_NaOBr	Li	0.64	0.55(8)	8.34 × 10^−8^	4.68 × 10^−4^
	Na	0.65	0.56(1)	8.38 × 10^−8^	4.97 × 10^−4^
Na_2_LiOCl	Li	0.80	0.72	2.57 × 10^−10^	1.87 × 10^−5^
	Na	0.64	0.57	6.84 × 10^−8^	4.64 × 10^−4^
Na_2_LiOBr	Li	0.74	0.66	5.90 × 10^−10^	1.66 × 10^−5^
	Na	0.67	0.59	1.77 × 10^−8^	1.67 × 10^−4^

The origin of the higher diffusion coefficient and conductivity in mixed Li_2_NaOX and Na_2_LiOX structures can be rationalized by the defect formation energy of Li/Na halide partial Schottky defects. For instance, the formation energy of Na-halide Schottky defect in Na_2_LiOCl and Na_2_LiOBr is lower as compared to their Li-halide counterparts.^16^ For Li_2_NaOX compounds, the Li/Na-X formation energy is similar, in accordance with the results on diffusion/conduction data depicted in Fig. 6 and 7. On the other hand, reports concerning the contradiction of conventional typecast are based on the ionic radius difference between Li (0.59 Å) and Na (0.99 Å).^28–30^ Considering the A^+^ ionic size as indicated by the mean ionic radius of Li and Na (*i.e. r*(A) = 0.79 Å), one can conclude that a substitution of Na occupying the A-site results as a consequence in a strong local strain/stress in the Li_2_NaOX and Na_2_LiOX structures, reducing the Na -V_A_ distance and the diffusion length. Besides, a Li occupancy of an A-site enlarges the Li- V_A_ distance. An A-halide Schottky defect leads to A-vacancies together with the competition between Li and Na atoms to reach the vacancies of A, and thereby constitutes the origin of the conductive behaviour in Li_2_NaOX and Na_2_LiOX structures.

**Fig. 8:**
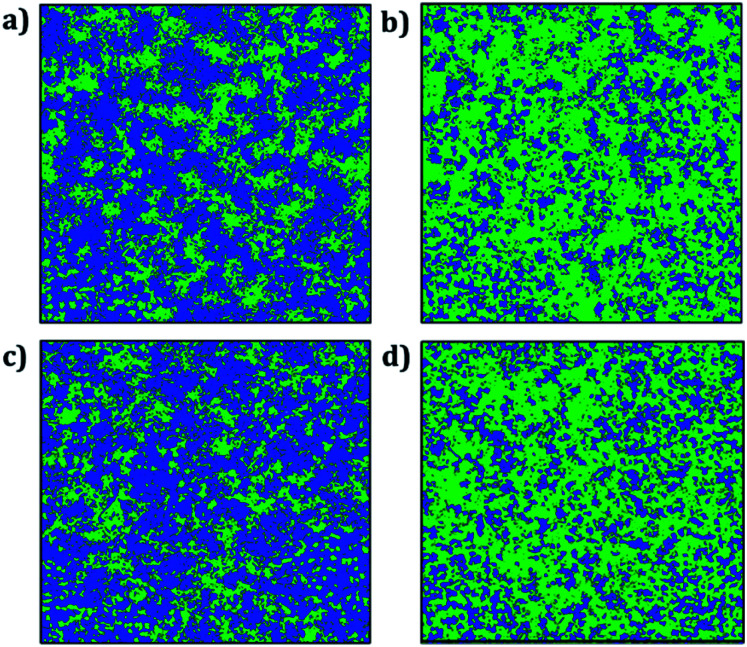
Trajectory plots of Li and Na in (a) Li_2_NaOCl, (b) Na_2_LiOCl, (c) Li_2_NaOBr and (d) Na_2_LiOBr compounds after 2 ns. Blue and green lines represent the Li and Na trajectory lines, respectively.

Fig. 8 displays the trajectory density plots of Li_2_NaOX and Na_2_LiOX structures at 2 ns. The density map profiles are similar to those discussed in the case of A_3_OX materials. The main difference consists in the fact that both Li and Na ion transports are favoured with marginally higher activation energies and lower conductivity at operative temperatures. Similar findings of individual alkali ion migration were discussed,^16^ where it was concluded that these mixed cation systems (*i.e.* Li_2_NaOX and Na_2_LiOX structures) are unfavourable to be used as inorganic electrolytes on the basis of the activation energy and diffusion/conduction at operative temperatures.

## 4. Conclusions

The influence of the presence of ∑3(111) grain boundaries on the transport properties of A_3_OX nanocrystalline samples was disclosed. Large scale molecular dynamic simulations showed that these nanocrystalline samples have higher activation energies as compared to values reported in previous theoretical studies, but much closer to the experimental data. This is attributed to the higher atomic density at the ∑3(111) grain boundaries. The pristine Na_3_OBr sample is characterized by the lower activation energies for Na-ion diffusion and conduction, making this compound promising for future solid state electrolyte in Na-ion batteries technologies. Despite some lowering of transport properties of the mixed samples Li_2_NaOX and Na_2_LiOX, they can also be considered as inorganic solid electrolytes in both Li- and Na-Ion batteries.

## Conflicts of interest

There are no conflicts to declare.

## Supplementary Material

## References

[cit1] Ahniyaz A., de Meatza I., Kvasha A., Garcia-Calvo O., Ahmed I., Sgroi M. F., Giuliano M., Dotoli M., Dumitrescu M. A., Jahn M., Zhang N. (2021). Adv. Appl. Energy.

[cit2] Zheng J., Wu Y., Sun Y., Rong J., Li H., Niu L. (2021). Nano-Micro Lett..

[cit3] Yi T. F., Wei T. T., Li Y., He Y. B., Wang Z. B. (2020). Energy Storage Mater..

[cit4] Fu Z., Chen X., Zhang Q. (2022). Wiley Interdiscip. Rev.: Comput. Mol. Sci..

[cit5] Xia W., Zhao Y., Zhao F., Adair K., Zhao R., Li S., Zou R., Zhao Y., Sun X. (2022). Chem. Rev..

[cit6] Franco A. A., Rucci A., Brandell D., Frayret C., Gaberscek M., Jankowski P., Johansson P. (2019). Chem. Rev..

[cit7] Ma X.-X., Shen X., Chen X., Fu Z.-H., Yao N., Zhang R., Zhang Q. (2022). Small Struct..

[cit8] Sagotra A. K., Cazorla C. (2017). ACS Appl. Mater. Interfaces.

[cit9] Lü X., Wu G., Howard J. W., Chen A., Zhao Y., Daemen L. L., Jia Q. (2014). Chem. Commun..

[cit10] Nguyen H., Hy S., Wu E., Deng Z., Samiee M., Yersak T., Luo J., Ong S. P., Meng Y. S. (2016). J. Electrochem. Soc..

[cit11] Wu M., Xu B., Lei X., Huang K., Ouyang C. (2018). J. Mater. Chem. A.

[cit12] Lü X., Howard J. W., Chen A., Zhu J., Li S., Wu G., Dowden P., Xu H., Zhao Y., Jia Q. (2016). Adv. Sci..

[cit13] Mouta R., Melo M. Á. B., Diniz E. M., Paschoal C. W. A. (2014). Chem. Mater..

[cit14] Zhu J., Li S., Zhang Y., Howard J. W., Lü X., Li Y., Wang Y., Kumar R. S., Wang L., Zhao Y. (2016). Appl. Phys. Lett..

[cit15] Dawson J. A., Canepa P., Famprikis T., Masquelier C., Islam M. S. (2018). J. Am. Chem. Soc..

[cit16] Dawson J. A., Chen H., Saiful Islam M. (2018). J. Phys. Chem. C.

[cit17] Zhu J., Wang Y., Li S., Howard J. W., Neuefeind J., Ren Y., Wang H., Liang C., Yang W., Zou R., Jin C., Zhao Y. (2016). Inorg. Chem..

[cit18] Zhao Y., Daemen L. L. (2012). J. Am. Chem. Soc..

[cit19] Chen B., Xu C., Zhou J. (2018). J. Electrochem. Soc..

[cit20] Sattar M. A., Javed M., Benkraouda M., Amrane N. (2021). Int. J. Energy Res..

[cit21] Hirel P. (2015). Comput. Phys. Commun..

[cit22] Plimpton S. (1995). J. Comput. Phys..

[cit23] Famprikis T., Canepa P., Dawson J. A., Islam M. S., Masquelier C. (2019). Nat. Mater..

[cit24] Zulueta Y. A., Nguyen M. T. (2021). Dalton Trans..

[cit25] Dawson J. A., Famprikis T., Johnston K. E. (2021). J. Mater. Chem. A.

[cit26] Heenen H. H., Voss J., Scheurer C., Reuter K., Luntz A. C. (2019). J. Phys. Chem. Lett..

[cit27] Li H. X., Zhou X. Y., Wang Y. C., Jiang H. (2021). Inorg. Chem. Front..

[cit28] Jung S. C., Kim H. J., Choi J. W., Han Y. K. (2014). Nano Lett..

[cit29] Ling C., Zhang R. (2017). Phys. Chem. Chem. Phys..

[cit30] Zulueta Y. A., Geerlings P., Tielens F., Nguyen M. T. (2019). J. Solid State Chem..

